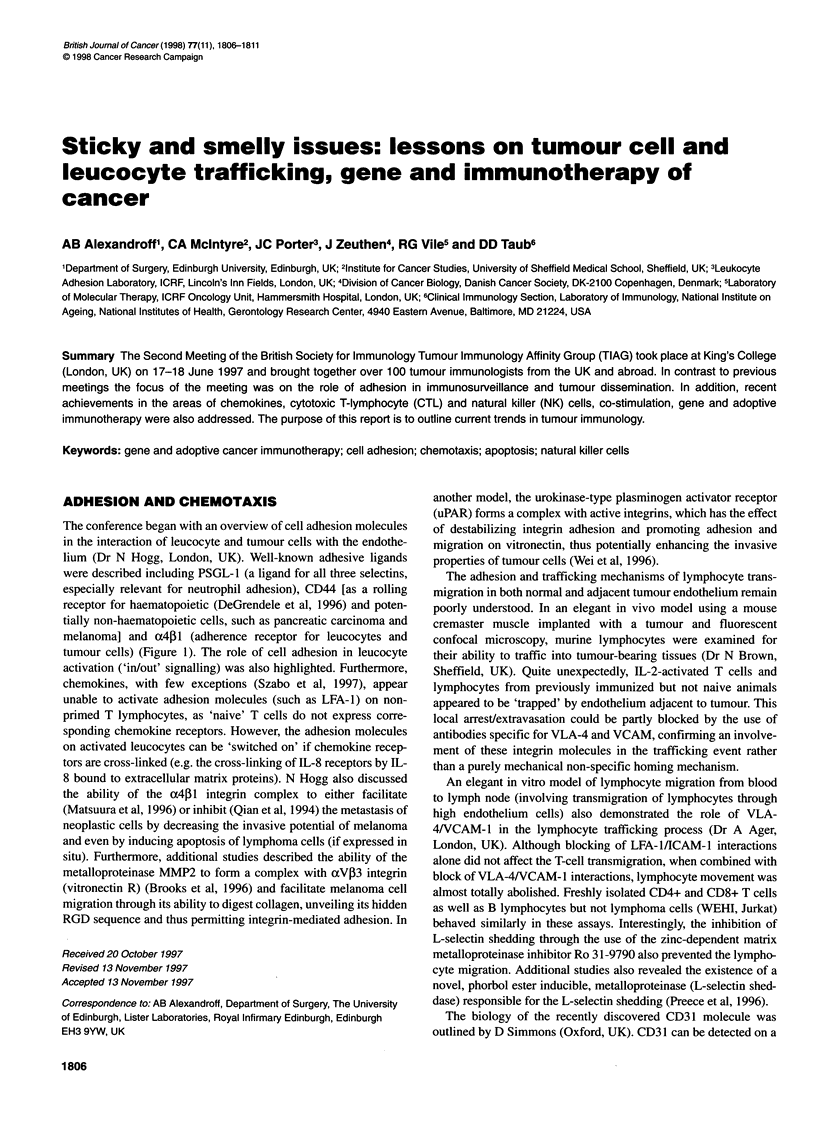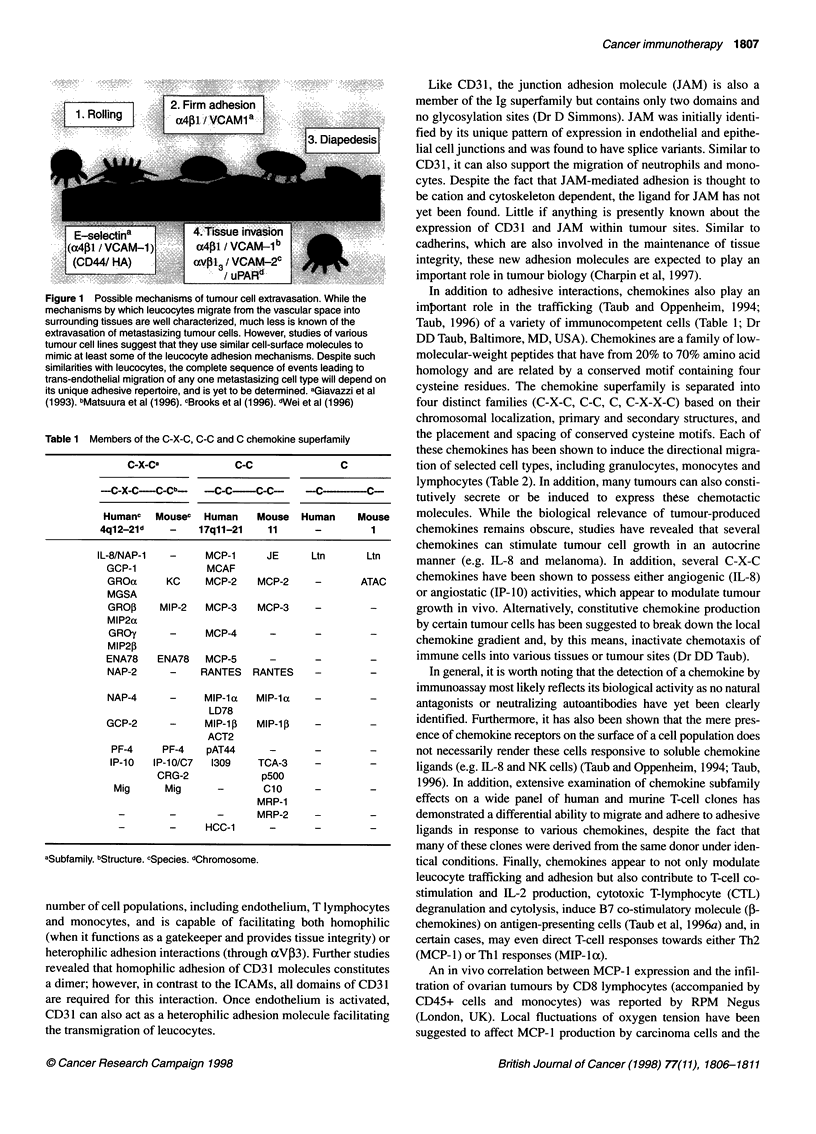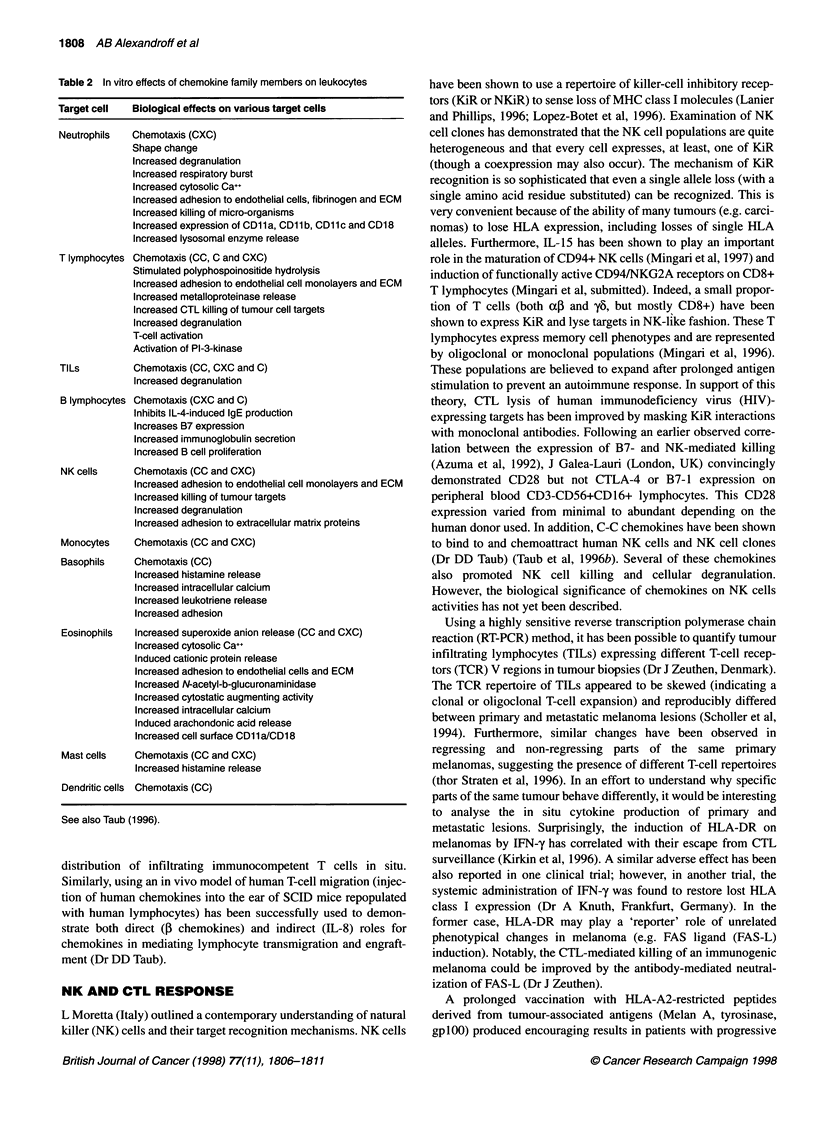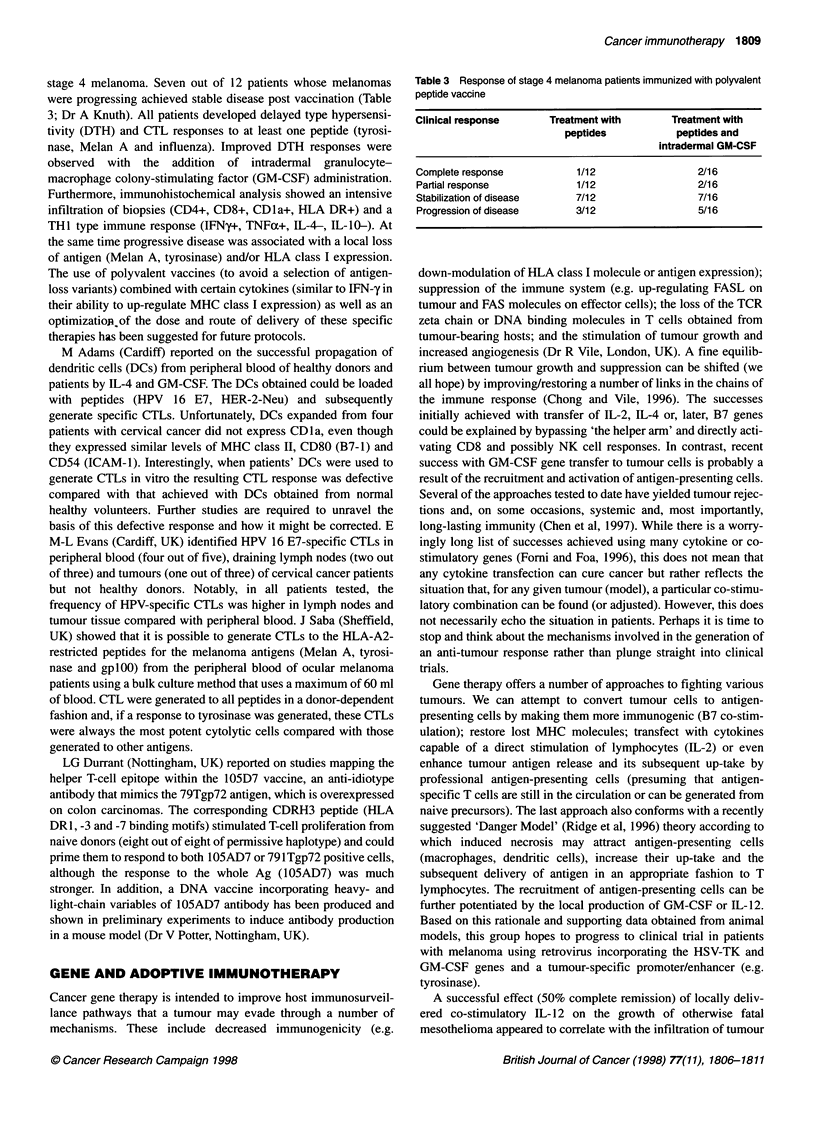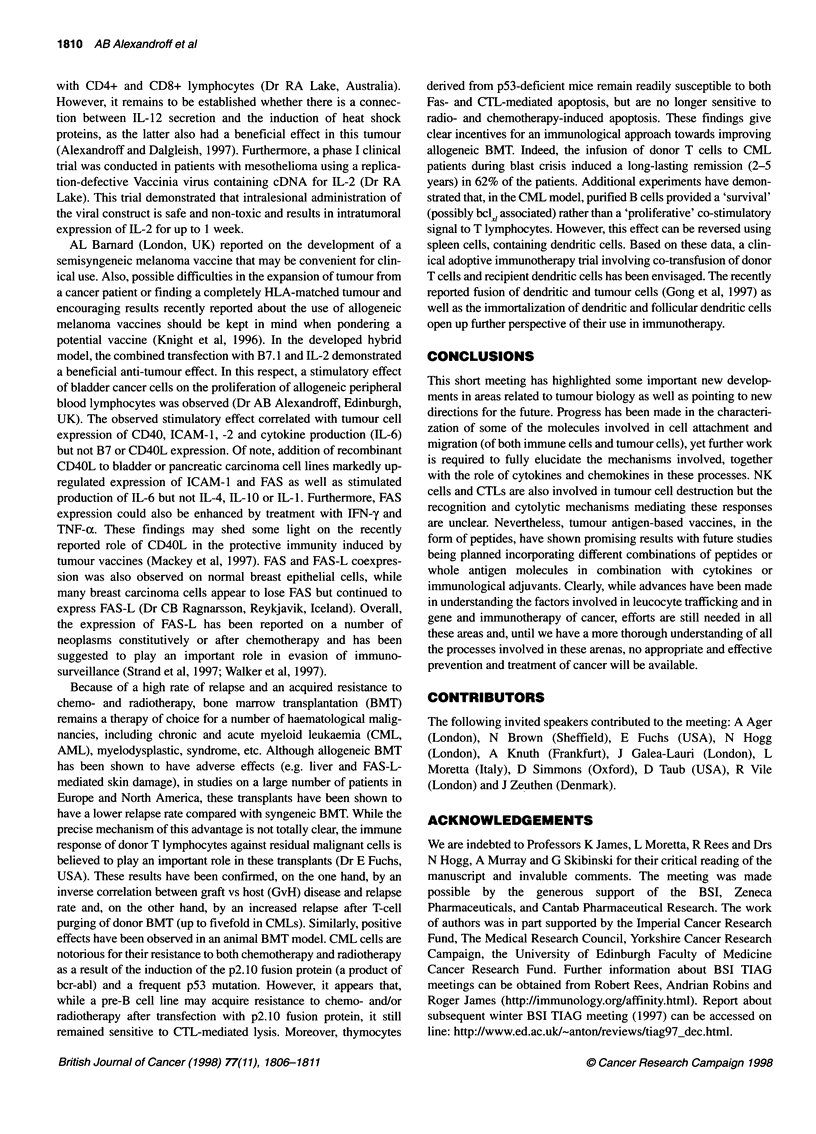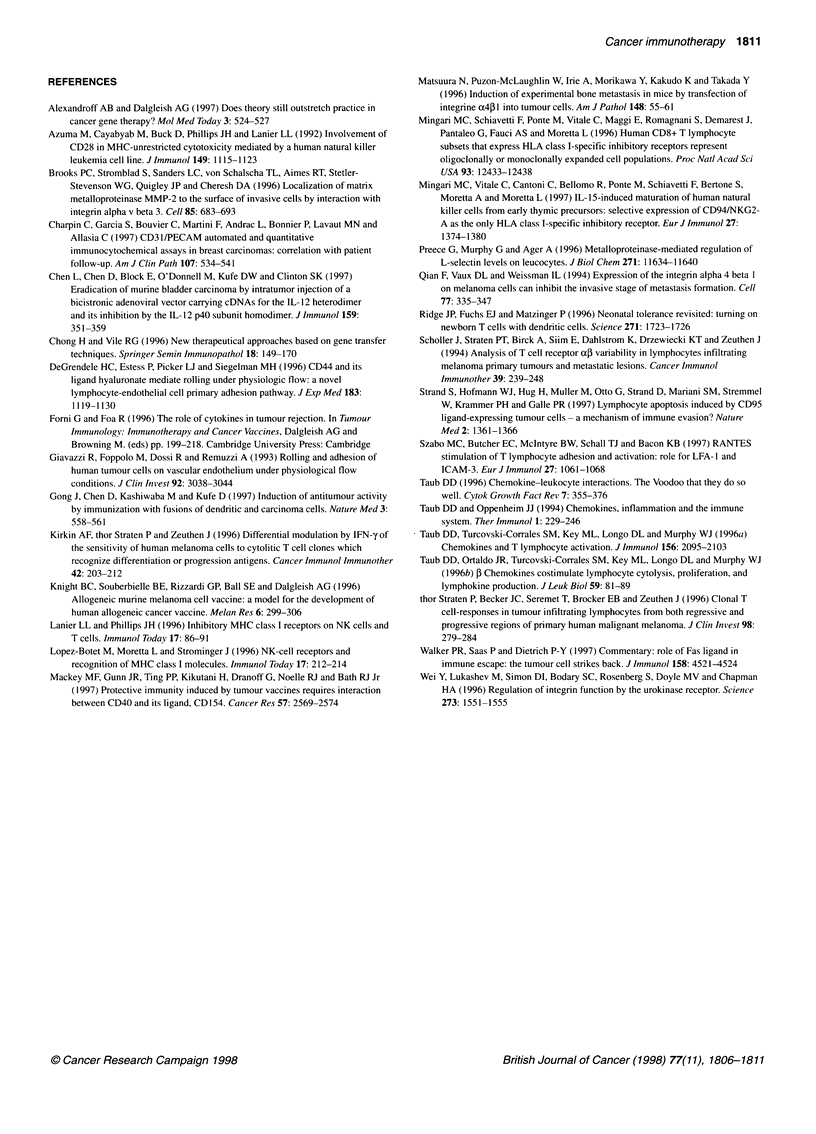# Sticky and smelly issues: lessons on tumour cell and leucocyte trafficking, gene and immunotherapy of cancer.

**DOI:** 10.1038/bjc.1998.300

**Published:** 1998-06

**Authors:** A. B. Alexandroff, C. A. McIntyre, J. C. Porter, J. Zeuthen, R. G. Vile, D. D. Taub

**Affiliations:** Department of Surgery, Edinburgh University, UK.

## Abstract

**Images:**


*British Journal of Cancer* (1998) **77**(11), 1806–1811
© 1998 Cancer Research Campaign

### Sticky and smelly issues: lessons on tumour cell and leucocyte trafficking, gene and immunotherapy of cancer

**AB Alexandroff[1], CA McIntyre[2], JC Porter[3], J Zeuthen[4], RG Vile[5] and DD Taub[6]**

[1]Department of Surgery, Edinburgh University, Edinburgh, UK; [2]Institute for Cancer Studies, University of Sheffield Medical School, Sheffield, UK; [3]Leukocyte Adhesion Laboratory, ICRF, Lincoln's Inn Fields, London, UK; [4]Division of Cancer Biology, Danish Cancer Society, DK-2100 Copenhagen, Denmark; [5]Laboratory of Molecular Therapy, ICRF Oncology Unit, Hammersmith Hospital, London, UK; [6]Clinical Immunology Section, Laboratory of Immunology, National Institute on Ageing, National Institutes of Health, Gerontology Research Center, 4940 Eastern Avenue, Baltimore, MD 21224, USA

**Summary** The Second Meeting of the British Society for Immunology Tumour Immunology Affinity Group (TIAG) took place at King's College (London, UK) on 17–18 June 1997 and brought together over 100 tumour immunologists from the UK and abroad. In contrast to previous meetings the focus of the meeting was on the role of adhesion in immunosurveillance and tumour dissemination. In addition, recent achievements in the areas of chemokines, cytotoxic T-lymphocyte (CTL) and natural killer (NK) cells, co-stimulation, gene and adoptive immunotherapy were also addressed. The purpose of this report is to outline current trends in tumour immunology.

**Keywords:** gene and adoptive cancer immunotherapy; cell adhesion; chemotaxis; apoptosis; natural killer cells

### ADHESION AND CHEMOTAXIS

The conference began with an overview of cell adhesion molecules in the interaction of leucocyte and tumour cells with the endothelium (Dr N Hogg, London, UK). Well-known adhesive ligands were described including PSGL-1 (a ligand for all three selectins, especially relevant for neutrophil adhesion), CD44 [as a rolling receptor for haematopoietic (DeGrendele et al, 1996) and potentially non-haematopoietic cells, such as pancreatic carcinoma and melanoma] and $\alpha4\beta1$ (adherence receptor for leucocytes and tumour cells) (Figure 1). The role of cell adhesion in leucocyte activation ('in/out' signalling) was also highlighted. Furthermore, chemokines, with few exceptions (Szabo et al, 1997), appear unable to activate adhesion molecules (such as LFA-1) on non-primed T lymphocytes, as 'naive' T cells do not express corresponding chemokine receptors. However, the adhesion molecules on activated leucocytes can be 'switched on' if chemokine receptors are cross-linked (e.g. the cross-linking of IL-8 receptors by IL-8 bound to extracellular matrix proteins). N Hogg also discussed the ability of the $\alpha4\beta1$ integrin complex to either facilitate (Matsuura et al, 1996) or inhibit (Qian et al, 1994) the metastasis of neoplastic cells by decreasing the invasive potential of melanoma and even by inducing apoptosis of lymphoma cells (if expressed in situ). Furthermore, additional studies described the ability of the metalloproteinase MMP2 to form a complex with $\alpha V\beta3$ integrin (vitronectin R) (Brooks et al, 1996) and facilitate melanoma cell migration through its ability to digest collagen, unveiling its hidden RGD sequence and thus permitting integrin-mediated adhesion. In another model, the urokinase-type plasminogen activator receptor (uPAR) forms a complex with active integrins, which has the effect of destabilizing integrin adhesion and promoting adhesion and migration on vitronectin, thus potentially enhancing the invasive properties of tumour cells (Wei et al, 1996).

The adhesion and trafficking mechanisms of lymphocyte transmigration in both normal and adjacent tumour endothelium remain poorly understood. In an elegant in vivo model using a mouse cremaster muscle implanted with a tumour and fluorescent confocal microscopy, murine lymphocytes were examined for their ability to traffic into tumour-bearing tissues (Dr N Brown, Sheffield, UK). Quite unexpectedly, IL-2-activated T cells and lymphocytes from previously immunized but not naive animals appeared to be 'trapped' by endothelium adjacent to tumour. This local arrest/extravasation could be partly blocked by the use of antibodies specific for VLA-4 and VCAM, confirming an involvement of these integrin molecules in the trafficking event rather than a purely mechanical non-specific homing mechanism.

An elegant in vitro model of lymphocyte migration from blood to lymph node (involving transmigration of lymphocytes through high endothelium cells) also demonstrated the role of VLA-4/VCAM-1 in the lymphocyte trafficking process (Dr A Ager, London, UK). Although blocking of LFA-1/ICAM-1 interactions alone did not affect the T-cell transmigration, when combined with block of VLA-4/VCAM-1 interactions, lymphocyte movement was almost totally abolished. Freshly isolated CD4+ and CD8+ T cells as well as B lymphocytes but not lymphoma cells (WEHI, Jurkat) behaved similarly in these assays. Interestingly, the inhibition of L-selectin shedding through the use of the zinc-dependent matrix metalloproteinase inhibitor Ro 31-9790 also prevented the lymphocyte migration. Additional studies also revealed the existence of a novel, phorbol ester inducible, metalloproteinase (L-selectin sheddase) responsible for the L-selectin shedding (Preece et al, 1996).

The biology of the recently discovered CD31 molecule was outlined by D Simmons (Oxford, UK). CD31 can be detected on a

*Received 20 October 1997*
*Revised 13 November 1997*
*Accepted 13 November 1997*

*Correspondence to:* AB Alexandroff, Department of Surgery, The University of Edinburgh, Lister Laboratories, Royal Infirmary Edinburgh, Edinburgh EH3 9YW, UK

**Figure 1** Possible mechanisms of tumour cell extravasation. While the mechanisms by which leucocytes migrate from the vascular space into surrounding tissues are well characterized, much less is known of the extravasation of metastasizing tumour cells. However, studies of various tumour cell lines suggest that they use similar cell-surface molecules to mimic at least some of the leucocyte adhesion mechanisms. Despite such similarities with leucocytes, the complete sequence of events leading to trans-endothelial migration of any one metastasizing cell type will depend on its unique adhesive repertoire, and is yet to be determined. [a]Giavazzi et al (1993). [b]Matsuura et al (1996). [c]Brooks et al (1996). [d]Wei et al (1996)

**Table 1** Members of the C-X-C, C-C and C chemokine superfamily

| C-X-C[a] | | C-C | | C | |
|---|---|---|---|---|---|
| ---C-X-C-----C-C[b]--- | | ---C-C-------C-C--- | | ---C-------------C--- | |
| Human[c] 4q12–21[d] | Mouse[c] – | Human 17q11–21 | Mouse 11 | Human – | Mouse 1 |
| IL-8/NAP-1 GCP-1 | – | MCP-1 MCAF | JE | Ltn | Ltn |
| GROα MGSA | KC | MCP-2 | MCP-2 | – | ATAC |
| GROβ MIP2α | MIP-2 | MCP-3 | MCP-3 | – | – |
| GROγ MIP2β | – | MCP-4 | – | – | – |
| ENA78 | ENA78 | MCP-5 | – | – | – |
| NAP-2 | – | RANTES | RANTES | – | – |
| NAP-4 | – | MIP-1α LD78 | MIP-1α | – | – |
| GCP-2 | – | MIP-1β ACT2 | MIP-1β | – | – |
| PF-4 | PF-4 | pAT44 | – | – | – |
| IP-10 | IP-10/C7 CRG-2 | I309 | TCA-3 p500 | – | – |
| Mig | Mig | – | C10 MRP-1 | – | – |
| – | – | – | MRP-2 | – | – |
| – | – | HCC-1 | – | – | – |

[a]Subfamily. [b]Structure. [c]Species. [d]Chromosome.

number of cell populations, including endothelium, T lymphocytes and monocytes, and is capable of facilitating both homophilic (when it functions as a gatekeeper and provides tissue integrity) or heterophilic adhesion interactions (through αVβ3). Further studies revealed that homophilic adhesion of CD31 molecules constitutes a dimer; however, in contrast to the ICAMs, all domains of CD31 are required for this interaction. Once endothelium is activated, CD31 can also act as a heterophilic adhesion molecule facilitating the transmigration of leucocytes.

Like CD31, the junction adhesion molecule (JAM) is also a member of the Ig superfamily but contains only two domains and no glycosylation sites (Dr D Simmons). JAM was initially identified by its unique pattern of expression in endothelial and epithelial cell junctions and was found to have splice variants. Similar to CD31, it can also support the migration of neutrophils and monocytes. Despite the fact that JAM-mediated adhesion is thought to be cation and cytoskeleton dependent, the ligand for JAM has not yet been found. Little if anything is presently known about the expression of CD31 and JAM within tumour sites. Similar to cadherins, which are also involved in the maintenance of tissue integrity, these new adhesion molecules are expected to play an important role in tumour biology (Charpin et al, 1997).

In addition to adhesive interactions, chemokines also play an important role in the trafficking (Taub and Oppenheim, 1994; Taub, 1996) of a variety of immunocompetent cells (Table 1; Dr DD Taub, Baltimore, MD, USA). Chemokines are a family of low-molecular-weight peptides that have from 20% to 70% amino acid homology and are related by a conserved motif containing four cysteine residues. The chemokine superfamily is separated into four distinct families (C-X-C, C-C, C, C-X-X-C) based on their chromosomal localization, primary and secondary structures, and the placement and spacing of conserved cysteine motifs. Each of these chemokines has been shown to induce the directional migration of selected cell types, including granulocytes, monocytes and lymphocytes (Table 2). In addition, many tumours can also constitutively secrete or be induced to express these chemotactic molecules. While the biological relevance of tumour-produced chemokines remains obscure, studies have revealed that several chemokines can stimulate tumour cell growth in an autocrine manner (e.g. IL-8 and melanoma). In addition, several C-X-C chemokines have been shown to possess either angiogenic (IL-8) or angiostatic (IP-10) activities, which appear to modulate tumour growth in vivo. Alternatively, constitutive chemokine production by certain tumour cells has been suggested to break down the local chemokine gradient and, by this means, inactivate chemotaxis of immune cells into various tissues or tumour sites (Dr DD Taub).

In general, it is worth noting that the detection of a chemokine by immunoassay most likely reflects its biological activity as no natural antagonists or neutralizing autoantibodies have yet been clearly identified. Furthermore, it has also been shown that the mere presence of chemokine receptors on the surface of a cell population does not necessarily render these cells responsive to soluble chemokine ligands (e.g. IL-8 and NK cells) (Taub and Oppenheim, 1994; Taub, 1996). In addition, extensive examination of chemokine subfamily effects on a wide panel of human and murine T-cell clones has demonstrated a differential ability to migrate and adhere to adhesive ligands in response to various chemokines, despite the fact that many of these clones were derived from the same donor under identical conditions. Finally, chemokines appear to not only modulate leucocyte trafficking and adhesion but also contribute to T-cell co-stimulation and IL-2 production, cytotoxic T-lymphocyte (CTL) degranulation and cytolysis, induce B7 co-stimulatory molecule (β-chemokines) on antigen-presenting cells (Taub et al, 1996*a*) and, in certain cases, may even direct T-cell responses towards either Th2 (MCP-1) or Th1 responses (MIP-1α).

An in vivo correlation between MCP-1 expression and the infiltration of ovarian tumours by CD8 lymphocytes (accompanied by CD45+ cells and monocytes) was reported by RPM Negus (London, UK). Local fluctuations of oxygen tension have been suggested to affect MCP-1 production by carcinoma cells and the

© Cancer Research Campaign 1998

**Table 2** In vitro effects of chemokine family members on leukocytes

| Target cell | Biological effects on various target cells |
|---|---|
| Neutrophils | Chemotaxis (CXC)<br>Shape change<br>Increased degranulation<br>Increased respiratory burst<br>Increased cytosolic $Ca^{++}$<br>Increased adhesion to endothelial cells, fibrinogen and ECM<br>Increased killing of micro-organisms<br>Increased expression of CD11a, CD11b, CD11c and CD18<br>Increased lysosomal enzyme release |
| T lymphocytes | Chemotaxis (CC, C and CXC)<br>Stimulated polyphospoinositide hydrolysis<br>Increased adhesion to endothelial cell monolayers and ECM<br>Increased metalloproteinase release<br>Increased CTL killing of tumour cell targets<br>Increased degranulation<br>T-cell activation<br>Activation of PI-3-kinase |
| TILs | Chemotaxis (CC, CXC and C)<br>Increased degranulation |
| B lymphocytes | Chemotaxis (CXC and C)<br>Inhibits IL-4-induced IgE production<br>Increases B7 expression<br>Increased immunoglobulin secretion<br>Increased B cell proliferation |
| NK cells | Chemotaxis (CC and CXC)<br>Increased adhesion to endothelial cell monolayers and ECM<br>Increased killing of tumour targets<br>Increased degranulation<br>Increased adhesion to extracellular matrix proteins |
| Monocytes | Chemotaxis (CC and CXC) |
| Basophils | Chemotaxis (CC)<br>Increased histamine release<br>Increased intracellular calcium<br>Increased leukotriene release<br>Increased adhesion |
| Eosinophils | Increased superoxide anion release (CC and CXC)<br>Increased cytosolic $Ca^{++}$<br>Induced cationic protein release<br>Increased adhesion to endothelial cells and ECM<br>Increased *N*-acetyl-b-glucuronaminidase<br>Increased cytostatic augmenting activity<br>Increased intracellular calcium<br>Induced arachondonic acid release<br>Increased cell surface CD11a/CD18 |
| Mast cells | Chemotaxis (CC and CXC)<br>Increased histamine release |
| Dendritic cells | Chemotaxis (CC) |

See also Taub (1996).

distribution of infiltrating immunocompetent T cells in situ. Similarly, using an in vivo model of human T-cell migration (injection of human chemokines into the ear of SCID mice repopulated with human lymphocytes) has been successfully used to demonstrate both direct (β chemokines) and indirect (IL-8) roles for chemokines in mediating lymphocyte transmigration and engraftment (Dr DD Taub).

### NK AND CTL RESPONSE

L Moretta (Italy) outlined a contemporary understanding of natural killer (NK) cells and their target recognition mechanisms. NK cells have been shown to use a repertoire of killer-cell inhibitory receptors (KiR or NKiR) to sense loss of MHC class I molecules (Lanier and Phillips, 1996; Lopez-Botet et al, 1996). Examination of NK cell clones has demonstrated that the NK cell populations are quite heterogeneous and that every cell expresses, at least, one of KiR (though a coexpression may also occur). The mechanism of KiR recognition is so sophisticated that even a single allele loss (with a single amino acid residue substituted) can be recognized. This is very convenient because of the ability of many tumours (e.g. carcinomas) to lose HLA expression, including losses of single HLA alleles. Furthermore, IL-15 has been shown to play an important role in the maturation of CD94+ NK cells (Mingari et al, 1997) and induction of functionally active CD94/NKG2A receptors on CD8+ T lymphocytes (Mingari et al, submitted). Indeed, a small proportion of T cells (both αβ and γδ, but mostly CD8+) have been shown to express KiR and lyse targets in NK-like fashion. These T lymphocytes express memory cell phenotypes and are represented by oligoclonal or monoclonal populations (Mingari et al, 1996). These populations are believed to expand after prolonged antigen stimulation to prevent an autoimmune response. In support of this theory, CTL lysis of human immunodeficiency virus (HIV)-expressing targets has been improved by masking KiR interactions with monoclonal antibodies. Following an earlier observed correlation between the expression of B7- and NK-mediated killing (Azuma et al, 1992), J Galea-Lauri (London, UK) convincingly demonstrated CD28 but not CTLA-4 or B7-1 expression on peripheral blood CD3-CD56+CD16+ lymphocytes. This CD28 expression varied from minimal to abundant depending on the human donor used. In addition, C-C chemokines have been shown to bind to and chemoattract human NK cells and NK cell clones (Dr DD Taub) (Taub et al, 1996*b*). Several of these chemokines also promoted NK cell killing and cellular degranulation. However, the biological significance of chemokines on NK cells activities has not yet been described.

Using a highly sensitive reverse transcription polymerase chain reaction (RT-PCR) method, it has been possible to quantify tumour infiltrating lymphocytes (TILs) expressing different T-cell receptors (TCR) V regions in tumour biopsies (Dr J Zeuthen, Denmark). The TCR repertoire of TILs appeared to be skewed (indicating a clonal or oligoclonal T-cell expansion) and reproducibly differed between primary and metastatic melanoma lesions (Scholler et al, 1994). Furthermore, similar changes have been observed in regressing and non-regressing parts of the same primary melanomas, suggesting the presence of different T-cell repertoires (thor Straten et al, 1996). In an effort to understand why specific parts of the same tumour behave differently, it would be interesting to analyse the in situ cytokine production of primary and metastatic lesions. Surprisingly, the induction of HLA-DR on melanomas by IFN-γ has correlated with their escape from CTL surveillance (Kirkin et al, 1996). A similar adverse effect has been also reported in one clinical trial; however, in another trial, the systemic administration of IFN-γ was found to restore lost HLA class I expression (Dr A Knuth, Frankfurt, Germany). In the former case, HLA-DR may play a 'reporter' role of unrelated phenotypical changes in melanoma (e.g. FAS ligand (FAS-L) induction). Notably, the CTL-mediated killing of an immunogenic melanoma could be improved by the antibody-mediated neutralization of FAS-L (Dr J Zeuthen).

A prolonged vaccination with HLA-A2-restricted peptides derived from tumour-associated antigens (Melan A, tyrosinase, gp100) produced encouraging results in patients with progressive

© Cancer Research Campaign 1998

stage 4 melanoma. Seven out of 12 patients whose melanomas were progressing achieved stable disease post vaccination (Table 3; Dr A Knuth). All patients developed delayed type hypersensitivity (DTH) and CTL responses to at least one peptide (tyrosinase, Melan A and influenza). Improved DTH responses were observed with the addition of intradermal granulocyte–macrophage colony-stimulating factor (GM-CSF) administration. Furthermore, immunohistochemical analysis showed an intensive infiltration of biopsies (CD4+, CD8+, CD1a+, HLA DR+) and a TH1 type immune response (IFNγ+, TNFα+, IL-4–, IL-10–). At the same time progressive disease was associated with a local loss of antigen (Melan A, tyrosinase) and/or HLA class I expression. The use of polyvalent vaccines (to avoid a selection of antigen-loss variants) combined with certain cytokines (similar to IFN-γ in their ability to up-regulate MHC class I expression) as well as an optimization of the dose and route of delivery of these specific therapies has been suggested for future protocols.

M Adams (Cardiff) reported on the successful propagation of dendritic cells (DCs) from peripheral blood of healthy donors and patients by IL-4 and GM-CSF. The DCs obtained could be loaded with peptides (HPV 16 E7, HER-2-Neu) and subsequently generate specific CTLs. Unfortunately, DCs expanded from four patients with cervical cancer did not express CD1a, even though they expressed similar levels of MHC class II, CD80 (B7-1) and CD54 (ICAM-1). Interestingly, when patients' DCs were used to generate CTLs in vitro the resulting CTL response was defective compared with that achieved with DCs obtained from normal healthy volunteers. Further studies are required to unravel the basis of this defective response and how it might be corrected. E M-L Evans (Cardiff, UK) identified HPV 16 E7-specific CTLs in peripheral blood (four out of five), draining lymph nodes (two out of three) and tumours (one out of three) of cervical cancer patients but not healthy donors. Notably, in all patients tested, the frequency of HPV-specific CTLs was higher in lymph nodes and tumour tissue compared with peripheral blood. J Saba (Sheffield, UK) showed that it is possible to generate CTLs to the HLA-A2-restricted peptides for the melanoma antigens (Melan A, tyrosinase and gp100) from the peripheral blood of ocular melanoma patients using a bulk culture method that uses a maximum of 60 ml of blood. CTL were generated to all peptides in a donor-dependent fashion and, if a response to tyrosinase was generated, these CTLs were always the most potent cytolytic cells compared with those generated to other antigens.

LG Durrant (Nottingham, UK) reported on studies mapping the helper T-cell epitope within the 105D7 vaccine, an anti-idiotype antibody that mimics the 79Tgp72 antigen, which is overexpressed on colon carcinomas. The corresponding CDRH3 peptide (HLA DR1, -3 and -7 binding motifs) stimulated T-cell proliferation from naive donors (eight out of eight of permissive haplotype) and could prime them to respond to both 105AD7 or 791Tgp72 positive cells, although the response to the whole Ag (105AD7) was much stronger. In addition, a DNA vaccine incorporating heavy- and light-chain variables of 105AD7 antibody has been produced and shown in preliminary experiments to induce antibody production in a mouse model (Dr V Potter, Nottingham, UK).

### GENE AND ADOPTIVE IMMUNOTHERAPY

Cancer gene therapy is intended to improve host immunosurveillance pathways that a tumour may evade through a number of mechanisms. These include decreased immunogenicity (e.g. down-modulation of HLA class I molecule or antigen expression); suppression of the immune system (e.g. up-regulating FASL on tumour and FAS molecules on effector cells); the loss of the TCR zeta chain or DNA binding molecules in T cells obtained from tumour-bearing hosts; and the stimulation of tumour growth and increased angiogenesis (Dr R Vile, London, UK). A fine equilibrium between tumour growth and suppression can be shifted (we all hope) by improving/restoring a number of links in the chains of the immune response (Chong and Vile, 1996). The successes initially achieved with transfer of IL-2, IL-4 or, later, B7 genes could be explained by bypassing 'the helper arm' and directly activating CD8 and possibly NK cell responses. In contrast, recent success with GM-CSF gene transfer to tumour cells is probably a result of the recruitment and activation of antigen-presenting cells. Several of the approaches tested to date have yielded tumour rejections and, on some occasions, systemic and, most importantly, long-lasting immunity (Chen et al, 1997). While there is a worryingly long list of successes achieved using many cytokine or co-stimulatory genes (Forni and Foa, 1996), this does not mean that any cytokine transfection can cure cancer but rather reflects the situation that, for any given tumour (model), a particular co-stimulatory combination can be found (or adjusted). However, this does not necessarily echo the situation in patients. Perhaps it is time to stop and think about the mechanisms involved in the generation of an anti-tumour response rather than plunge straight into clinical trials.

Gene therapy offers a number of approaches to fighting various tumours. We can attempt to convert tumour cells to antigen-presenting cells by making them more immunogenic (B7 co-stimulation); restore lost MHC molecules; transfect with cytokines capable of a direct stimulation of lymphocytes (IL-2) or even enhance tumour antigen release and its subsequent up-take by professional antigen-presenting cells (presuming that antigen-specific T cells are still in the circulation or can be generated from naive precursors). The last approach also conforms with a recently suggested 'Danger Model' (Ridge et al, 1996) theory according to which induced necrosis may attract antigen-presenting cells (macrophages, dendritic cells), increase their up-take and the subsequent delivery of antigen in an appropriate fashion to T lymphocytes. The recruitment of antigen-presenting cells can be further potentiated by the local production of GM-CSF or IL-12. Based on this rationale and supporting data obtained from animal models, this group hopes to progress to clinical trial in patients with melanoma using retrovirus incorporating the HSV-TK and GM-CSF genes and a tumour-specific promoter/enhancer (e.g. tyrosinase).

A successful effect (50% complete remission) of locally delivered co-stimulatory IL-12 on the growth of otherwise fatal mesothelioma appeared to correlate with the infiltration of tumour

**Table 3** Response of stage 4 melanoma patients immunized with polyvalent peptide vaccine

| Clinical response | Treatment with peptides | Treatment with peptides and intradermal GM-CSF |
|---|---|---|
| Complete response | 1/12 | 2/16 |
| Partial response | 1/12 | 2/16 |
| Stabilization of disease | 7/12 | 7/16 |
| Progression of disease | 3/12 | 5/16 |

© Cancer Research Campaign 1998

with CD4+ and CD8+ lymphocytes (Dr RA Lake, Australia). However, it remains to be established whether there is a connection between IL-12 secretion and the induction of heat shock proteins, as the latter also had a beneficial effect in this tumour (Alexandroff and Dalgleish, 1997). Furthermore, a phase I clinical trial was conducted in patients with mesothelioma using a replication-defective Vaccinia virus containing cDNA for IL-2 (Dr RA Lake). This trial demonstrated that intralesional administration of the viral construct is safe and non-toxic and results in intratumoral expression of IL-2 for up to 1 week.

AL Barnard (London, UK) reported on the development of a semisyngeneic melanoma vaccine that may be convenient for clinical use. Also, possible difficulties in the expansion of tumour from a cancer patient or finding a completely HLA-matched tumour and encouraging results recently reported about the use of allogeneic melanoma vaccines should be kept in mind when pondering a potential vaccine (Knight et al, 1996). In the developed hybrid model, the combined transfection with B7.1 and IL-2 demonstrated a beneficial anti-tumour effect. In this respect, a stimulatory effect of bladder cancer cells on the proliferation of allogeneic peripheral blood lymphocytes was observed (Dr AB Alexandroff, Edinburgh, UK). The observed stimulatory effect correlated with tumour cell expression of CD40, ICAM-1, -2 and cytokine production (IL-6) but not B7 or CD40L expression. Of note, addition of recombinant CD40L to bladder or pancreatic carcinoma cell lines markedly up-regulated expression of ICAM-1 and FAS as well as stimulated production of IL-6 but not IL-4, IL-10 or IL-1. Furthermore, FAS expression could also be enhanced by treatment with IFN-$\gamma$ and TNF-$\alpha$. These findings may shed some light on the recently reported role of CD40L in the protective immunity induced by tumour vaccines (Mackey et al, 1997). FAS and FAS-L coexpression was also observed on normal breast epithelial cells, while many breast carcinoma cells appear to lose FAS but continued to express FAS-L (Dr CB Ragnarsson, Reykjavik, Iceland). Overall, the expression of FAS-L has been reported on a number of neoplasms constitutively or after chemotherapy and has been suggested to play an important role in evasion of immunosurveillance (Strand et al, 1997; Walker et al, 1997).

Because of a high rate of relapse and an acquired resistance to chemo- and radiotherapy, bone marrow transplantation (BMT) remains a therapy of choice for a number of haematological malignancies, including chronic and acute myeloid leukaemia (CML, AML), myelodysplastic, syndrome, etc. Although allogeneic BMT has been shown to have adverse effects (e.g. liver and FAS-L-mediated skin damage), in studies on a large number of patients in Europe and North America, these transplants have been shown to have a lower relapse rate compared with syngeneic BMT. While the precise mechanism of this advantage is not totally clear, the immune response of donor T lymphocytes against residual malignant cells is believed to play an important role in these transplants (Dr E Fuchs, USA). These results have been confirmed, on the one hand, by an inverse correlation between graft vs host (GvH) disease and relapse rate and, on the other hand, by an increased relapse after T-cell purging of donor BMT (up to fivefold in CMLs). Similarly, positive effects have been observed in an animal BMT model. CML cells are notorious for their resistance to both chemotherapy and radiotherapy as a result of the induction of the p2.10 fusion protein (a product of bcr-abl) and a frequent p53 mutation. However, it appears that, while a pre-B cell line may acquire resistance to chemo- and/or radiotherapy after transfection with p2.10 fusion protein, it still remained sensitive to CTL-mediated lysis. Moreover, thymocytes derived from p53-deficient mice remain readily susceptible to both Fas- and CTL-mediated apoptosis, but are no longer sensitive to radio- and chemotherapy-induced apoptosis. These findings give clear incentives for an immunological approach towards improving allogeneic BMT. Indeed, the infusion of donor T cells to CML patients during blast crisis induced a long-lasting remission (2–5 years) in 62% of the patients. Additional experiments have demonstrated that, in the CML model, purified B cells provided a 'survival' (possibly $bcl_{xl}$ associated) rather than a 'proliferative' co-stimulatory signal to T lymphocytes. However, this effect can be reversed using spleen cells, containing dendritic cells. Based on these data, a clinical adoptive immunotherapy trial involving co-transfusion of donor T cells and recipient dendritic cells has been envisaged. The recently reported fusion of dendritic and tumour cells (Gong et al, 1997) as well as the immortalization of dendritic and follicular dendritic cells open up further perspective of their use in immunotherapy.

### CONCLUSIONS

This short meeting has highlighted some important new developments in areas related to tumour biology as well as pointing to new directions for the future. Progress has been made in the characterization of some of the molecules involved in cell attachment and migration (of both immune cells and tumour cells), yet further work is required to fully elucidate the mechanisms involved, together with the role of cytokines and chemokines in these processes. NK cells and CTLs are also involved in tumour cell destruction but the recognition and cytolytic mechanisms mediating these responses are unclear. Nevertheless, tumour antigen-based vaccines, in the form of peptides, have shown promising results with future studies being planned incorporating different combinations of peptides or whole antigen molecules in combination with cytokines or immunological adjuvants. Clearly, while advances have been made in understanding the factors involved in leucocyte trafficking and in gene and immunotherapy of cancer, efforts are still needed in all these areas and, until we have a more thorough understanding of all the processes involved in these arenas, no appropriate and effective prevention and treatment of cancer will be available.

### CONTRIBUTORS

The following invited speakers contributed to the meeting: A Ager (London), N Brown (Sheffield), E Fuchs (USA), N Hogg (London), A Knuth (Frankfurt), J Galea-Lauri (London), L Moretta (Italy), D Simmons (Oxford), D Taub (USA), R Vile (London) and J Zeuthen (Denmark).

### ACKNOWLEDGEMENTS

We are indebted to Professors K James, L Moretta, R Rees and Drs N Hogg, A Murray and G Skibinski for their critical reading of the manuscript and invaluble comments. The meeting was made possible by the generous support of the BSI, Zeneca Pharmaceuticals, and Cantab Pharmaceutical Research. The work of authors was in part supported by the Imperial Cancer Research Fund, The Medical Research Council, Yorkshire Cancer Research Campaign, the University of Edinburgh Faculty of Medicine Cancer Research Fund. Further information about BSI TIAG meetings can be obtained from Robert Rees, Andrian Robins and Roger James (http://immunology.org/affinity.html). Report about subsequent winter BSI TIAG meeting (1997) can be accessed on line: http://www.ed.ac.uk/~anton/reviews/tiag97_dec.html.

© Cancer Research Campaign 1998

### REFERENCES

Alexandroff AB and Dalgleish AG (1997) Does theory still outstretch practice in cancer gene therapy? *Mol Med Today* **3**: 524–527

Azuma M, Cayabyab M, Buck D, Phillips JH and Lanier LL (1992) Involvement of CD28 in MHC-unrestricted cytotoxicity mediated by a human natural killer leukemia cell line. *J Immunol* **149**: 1115–1123

Brooks PC, Stromblad S, Sanders LC, von Schalscha TL, Aimes RT, Stetler-Stevenson WG, Quigley JP and Cheresh DA (1996) Localization of matrix metalloproteinase MMP-2 to the surface of invasive cells by interaction with integrin alpha v beta 3. *Cell* **85**: 683–693

Charpin C, Garcia S, Bouvier C, Martini F, Andrac L, Bonnier P, Lavaut MN and Allasia C (1997) CD31/PECAM automated and quantitative immunocytochemical assays in breast carcinomas: correlation with patient follow-up. *Am J Clin Path* **107**: 534–541

Chen L, Chen D, Block E, O'Donnell M, Kufe DW and Clinton SK (1997) Eradication of murine bladder carcinoma by intratumor injection of a bicistronic adenoviral vector carrying cDNAs for the IL-12 heterodimer and its inhibition by the IL-12 p40 subunit homodimer. *J Immunol* **159**: 351–359

Chong H and Vile RG (1996) New therapeutical approaches based on gene transfer techniques. *Springer Semin Immunopathol* **18**: 149–170

DeGrendele HC, Estess P, Picker LJ and Siegelman MH (1996) CD44 and its ligand hyaluronate mediate rolling under physiologic flow: a novel lymphocyte-endothelial cell primary adhesion pathway. *J Exp Med* **183**: 1119–1130

Forni G and Foa R (1996) The role of cytokines in tumour rejection. In *Tumour Immunology: Immunotherapy and Cancer Vaccines*, Dalgleish AG and Browning M. (eds) pp. 199–218. Cambridge University Press: Cambridge

Giavazzi R, Foppolo M, Dossi R and Remuzzi A (1993) Rolling and adhesion of human tumour cells on vascular endothelium under physiological flow conditions. *J Clin Invest* **92**: 3038–3044

Gong J, Chen D, Kashiwaba M and Kufe D (1997) Induction of antitumour activity by immunization with fusions of dendritic and carcinoma cells. *Nature Med* **3**: 558–561

Kirkin AF, thor Straten P and Zeuthen J (1996) Differential modulation by IFN-γ of the sensitivity of human melanoma cells to cytolitic T cell clones which recognize differentiation or progression antigens. *Cancer Immunol Immunother* **42**: 203–212

Knight BC, Souberbielle BE, Rizzardi GP, Ball SE and Dalgleish AG (1996) Allogeneic murine melanoma cell vaccine: a model for the development of human allogeneic cancer vaccine. *Melan Res* **6**: 299–306

Lanier LL and Phillips JH (1996) Inhibitory MHC class I receptors on NK cells and T cells. *Immunol Today* **17**: 86–91

Lopez-Botet M, Moretta L and Strominger J (1996) NK-cell receptors and recognition of MHC class I molecules. *Immunol Today* **17**: 212–214

Mackey MF, Gunn JR, Ting PP, Kikutani H, Dranoff G, Noelle RJ and Bath RJ Jr (1997) Protective immunity induced by tumour vaccines requires interaction between CD40 and its ligand, CD154. *Cancer Res* **57**: 2569–2574

Matsuura N, Puzon-McLaughlin W, Irie A, Morikawa Y, Kakudo K and Takada Y (1996) Induction of experimental bone metastasis in mice by transfection of integrine α4β1 into tumour cells. *Am J Pathol* **148**: 55–61

Mingari MC, Schiavetti F, Ponte M, Vitale C, Maggi E, Romagnani S, Demarest J, Pantaleo G, Fauci AS and Moretta L (1996) Human CD8+ T lymphocyte subsets that express HLA class I-specific inhibitory receptors represent oligoclonally or monoclonally expanded cell populations. *Proc Natl Acad Sci USA* **93**: 12433–12438

Mingari MC, Vitale C, Cantoni C, Bellomo R, Ponte M, Schiavetti F, Bertone S, Moretta A and Moretta L (1997) IL-15-induced maturation of human natural killer cells from early thymic precursors: selective expression of CD94/NKG2-A as the only HLA class I-specific inhibitory receptor. *Eur J Immunol* **27**: 1374–1380

Preece G, Murphy G and Ager A (1996) Metalloproteinase-mediated regulation of L-selectin levels on leucocytes. *J Biol Chem* **271**: 11634–11640

Qian F, Vaux DL and Weissman IL (1994) Expression of the integrin alpha 4 beta 1 on melanoma cells can inhibit the invasive stage of metastasis formation. *Cell* **77**: 335–347

Ridge JP, Fuchs EJ and Matzinger P (1996) Neonatal tolerance revisited: turning on newborn T cells with dendritic cells. *Science* **271**: 1723–1726

Scholler J, Straten PT, Birck A, Siim E, Dahlstrom K, Drzewiecki KT and Zeuthen J (1994) Analysis of T cell receptor αβ variability in lymphocytes infiltrating melanoma primary tumours and metastatic lesions. *Cancer Immunol Immunother* **39**: 239–248

Strand S, Hofmann WJ, Hug H, Muller M, Otto G, Strand D, Mariani SM, Stremmel W, Krammer PH and Galle PR (1997) Lymphocyte apoptosis induced by CD95 ligand-expressing tumour cells – a mechanism of immune evasion? *Nature Med* **2**: 1361–1366

Szabo MC, Butcher EC, McIntyre BW, Schall TJ and Bacon KB (1997) RANTES stimulation of T lymphocyte adhesion and activation: role for LFA-1 and ICAM-3. *Eur J Immunol* **27**: 1061–1068

Taub DD (1996) Chemokine–leukocyte interactions. The Voodoo that they do so well. *Cytok Growth Fact Rev* **7**: 355–376

Taub DD and Oppenheim JJ (1994) Chemokines, inflammation and the immune system. *Ther Immunol* **1**: 229–246

Taub DD, Turcovski-Corrales SM, Key ML, Longo DL and Murphy WJ (1996*a*) Chemokines and T lymphocyte activation. *J Immunol* **156**: 2095–2103

Taub DD, Ortaldo JR, Turcovski-Corrales SM, Key ML, Longo DL and Murphy WJ (1996*b*) β Chemokines costimulate lymphocyte cytolysis, proliferation, and lymphokine production. *J Leuk Biol* **59**: 81–89

thor Straten P, Becker JC, Seremet T, Brocker EB and Zeuthen J (1996) Clonal T cell-responses in tumour infiltrating lymphocytes from both regressive and progressive regions of primary human malignant melanoma. *J Clin Invest* **98**: 279–284

Walker PR, Saas P and Dietrich P-Y (1997) Commentary: role of Fas ligand in immune escape: the tumour cell strikes back. *J Immunol* **158**: 4521–4524

Wei Y, Lukashev M, Simon DI, Bodary SC, Rosenberg S, Doyle MV and Chapman HA (1996) Regulation of integrin function by the urokinase receptor. *Science* **273**: 1551–1555

© Cancer Research Campaign 1998