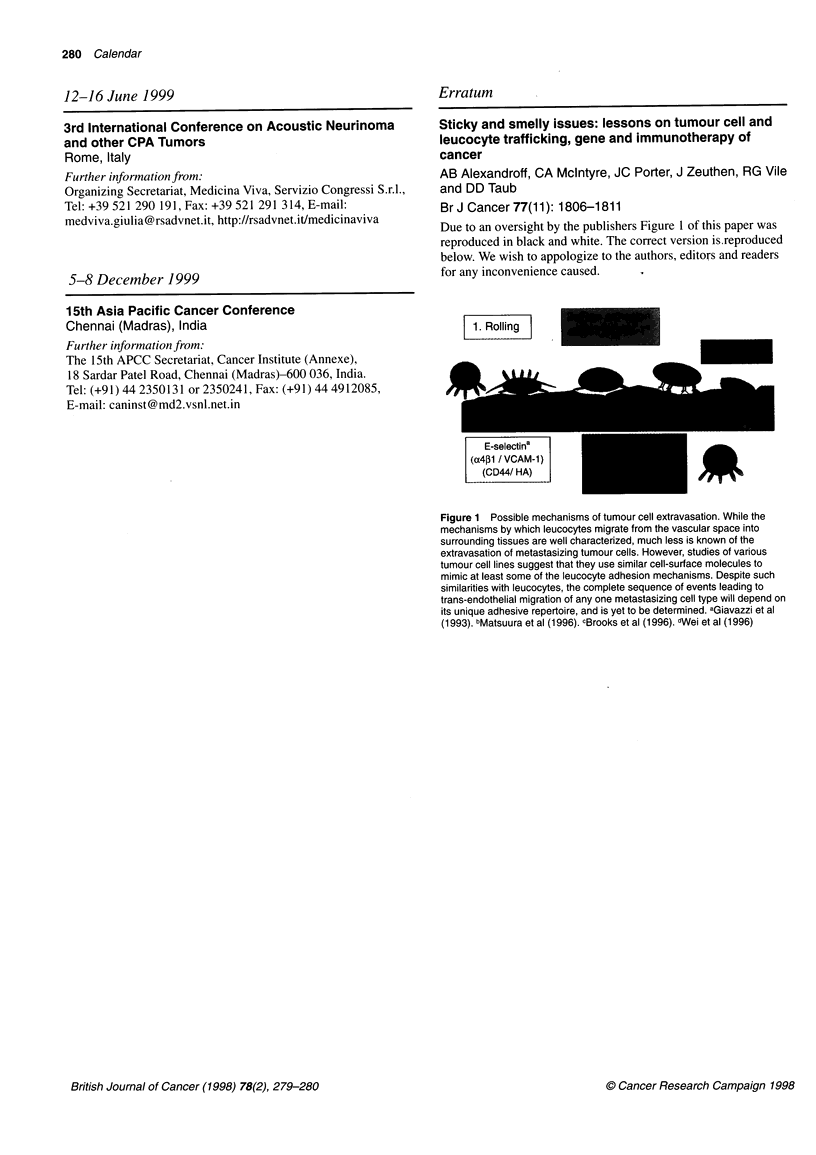# Sticky and smelly issues: lessons on tumour cell and leucocyte trafficking, gene and immunotherapy of cancer

**Published:** 1998-07

**Authors:** 

## Abstract

**Images:**


### *Erratum*

### Sticky and smelly issues: lessons on tumour cell and leucocyte trafficking, gene and immunotherapy of cancer

AB Alexandroff, CA McIntyre, JC Porter, J Zeuthen, RG Vile and DD Taub

Br J Cancer **77**(11): 1806–1811

Due to an oversight by the publishers Figure 1 of this paper was reproduced in black and white. The correct version is reproduced below. We wish to appologize to the authors, editors and readers for any inconvenience caused.

**Figure 1** Possible mechanisms of tumour cell extravasation. While the mechanisms by which leucocytes migrate from the vascular space into surrounding tissues are well characterized, much less is known of the extravasation of metastasizing tumour cells. However, studies of various tumour cell lines suggest that they use similar cell-surface molecules to mimic at least some of the leucocyte adhesion mechanisms. Despite such similarities with leucocytes, the complete sequence of events leading to trans-endothelial migration of any one metastasizing cell type will depend on its unique adhesive repertoire, and is yet to be determined. [a]Giavazzi et al (1993). [b]Matsuura et al (1996). [c]Brooks et al (1996). [d]Wei et al (1996)

*© Cancer Research Campaign 1998*